# The safety, efficacy, and functional outcomes on arthroscopic fixation of posterior cruciate ligament avulsion fracture by a bio-absorbable anchor or traditional pull-out technique: A prospective cohort study

**DOI:** 10.3389/fbioe.2022.1055176

**Published:** 2022-11-16

**Authors:** Xiangyu Ren, Jianing Wang, Shulong Yang, Zhe Liu, Tianda Wang, Teng Zhang, Haoxin Li, Zhong Zhang

**Affiliations:** ^1^ Department of Sports Medicine, The People’s Hospital of Wu Hai Inner Mongolia, Wuhai, China; ^2^ Department of Sports Medicine, Peking University Third Hospital. Institute of Sports Medicine of Peking University. Beijing Key Laboratory of Sports Injuries, Beijing, China

**Keywords:** posterior cruciate ligament avulsion fracture, bio-absorbable anchor, bio-material, safety, efficacy, functional outcome

## Abstract

**Background:** The posterior cruciate ligament avulsion fracture (PCLAF) is a special type of PCL rupture, and arthroscopic fixation for PCLAF has been recommended currently. The bio-absorbable suture anchor is a novel internal fixation for PCLAF. This study aims to estimate and compare the safety, efficacy, and functional outcomes between the bio-absorbable anchor and the traditional suture pull-out technique for arthroscopic fixation of PCLAF.

**Methods:** This was a prospective cohort study. PCLAF patients were included from 1 January 2020, to 31 August 2021, in our department, and randomly divided into the absorbable anchor group and control group (pull-out suture fixation). Clinical assessments included: post drawer test, gravity test, anterior-posterior laxity (KT-2000), range of motion, Lysholm and International Knee Documentation Committee (IKDC) scores, total failure rate, and returning to sports rate. The minimum follow-up was 1 year (y).

**Results:** 31 patients had accomplished the 1 year follow-up (missing rate: 13.9%). We did not face any complications such as neurovascular injury, fever, infection, un-union, or re-rupture during the follow-up. CT scan showed that all of the patients in the two groups had a well bone union at 3 months in post-operation. At 1 year follow-up, the total failure rate of the bio-absorbable anchor group (1/17, *p* = 0.036) was lower than the control group (5/14), and the IKDC (86.24 ± 4.35, *p* = 0.008) and return to sports rate (11/17, *p* = 0.045) of the bio-absorbable anchor group were higher than that of the control group (81.43 ± 5.06) (4/14).

**Conclusion:** Both the bio-absorbable anchor and suture pull-out technique for arthroscopic fixation of PCLAF have acquired a well bone union and superior safety, but the bio-absorbable anchor group had better efficacy and functional outcomes than the traditional pull-out technique.

## 1 Introduction

The posterior cruciate ligament (PCL) is a main stabilizer of knee, maintaining the rotation stability and posterior stability during motion (Rosenthal et al., 2012). PCL avulsion fracture (PCLAF) is a special type of PCL rupture, which mostly occurs at its tibial insertion. As the PCL ligament is usually intact, a reduction and fixation operation for reconstructing the tendon-bone integrity can acquire a good efficacy and functional outcome in patients with a fresh PCLAF (Zhang et al., 2013; Sabat et al., 2016). Currently, arthroscopic operations of PCLAF have been recommended, because of the advantages of mini-invasive, reliable reduction, ease of operation, and less complication (Domnick et al., 2016; Nourbakhsh et al., 2016; Hooper et al., 2017).

Recently, the biomaterial science development has greatly advanced the arthroscopic fixation techniques of PCLAF. One of the most representative biomaterial fixations is the bio-absorbable suture anchor, which can be used to perform the arthroscopic “suture bridge” fixation for PCLAF. The suture bridge technique has been commonly used in arthroscopic repair of the rotator cuff, it is easy to perform, and has been described as an effective method for obtaining higher initial fixation strength, larger contact area, and higher contact pressure at the tendinous footprint, compared with the traditional former techniques (Kim et al., 2006). The novel bio-absorbable can produce a rigid fixation for PCLAF. The main component of the bio-absorbable suture anchor is polylactide, which melts over the suture during the fixation process, creating an inextricable connection between the anchor and suture, as well as a rigid fixation between the anchor and bone (Koch et al., 2021). What’s more, Similar to the traditional pull-out suture fixation, the bio-absorbable suture anchor is also applicable to PCLAF with small or comminuted fragments (Willinger et al., 2019) (Nakagawa et al., 2017).

The clinical application of the novel bio-absorbable anchor has been paid more and more attention in the fields of biomaterial science and sports medicine. The first arthroscopic suture bridge fixation of PCLAF (non-absorbable) was reported in 2016 (Nourbakhsh et al., 2016), till now, clinical study on using the bio-absorbable suture anchor in this field is still limited, and only several cases were reported, lacking of systematic follow-up study and clinical comparisons. The purpose of this study is to estimate and compare the safety, efficacy, and functional outcomes of arthroscopic fixation of PCLAF between the bio-absorbable suture anchor and the traditional technique.

## 2 Materials and methods

### 2.1 Patient involvement

Patients diagnosed with PCLAF were included between 1 January 2020, and 31 August 2021, in our department. The inclusion criteria were: 1) age: 18–39 years old, BMI≤31; 2) acute PCLAF (injury less than 3 weeks) (Madi et al., 2021); 3) MRI showed a definite PCLAF with partial, complete, or comminuted fragment; 4) isolated PCLAF in a single knee; 5) agree to participate in this study after signing the informed consent; 6) agree to the arrangement of the grouping. The exclusion criteria were: 1) the periarticular fractures; 2) PCL re-rupture; 3) patients combined with ACL rupture, MCL rupture, or meniscus tears; 4) knee osteoarthritis with the Kellgren-Lawrence grade>2; 5) systematic diseases such as rheumatoid arthritis, gouty arthritis, nerve system diseases, diabetes.

Patients were randomly divided into the absorbable anchor group and control group (pull-out suture fixation). The protocol and procedure for protecting human subjects in the present study were approved by the Ethics Committee (IRB ethical approval: KS-ob202171) in our hospital before this study started.

### 2.2 Arthroscopic fixation of PCLAF with bio-absorbable anchor or pull-out technique

Operations of the two groups were performed by the same senior surgeon, with the patients supine, under spinal anesthesia, and using a tourniquet. A standard anterolateral (AL) and anteromedial (AM) portals were made, and an initial diagnostic arthroscopy and debridement were carried out until the posteromedial (PM) compartment is visualized, and then a high PM portal (arthroscope) and a low PM (working) portal were created by a guide needle. After identifying the bone fragment, debridement of the fracture bed was performed.

**FIGURE 1:**
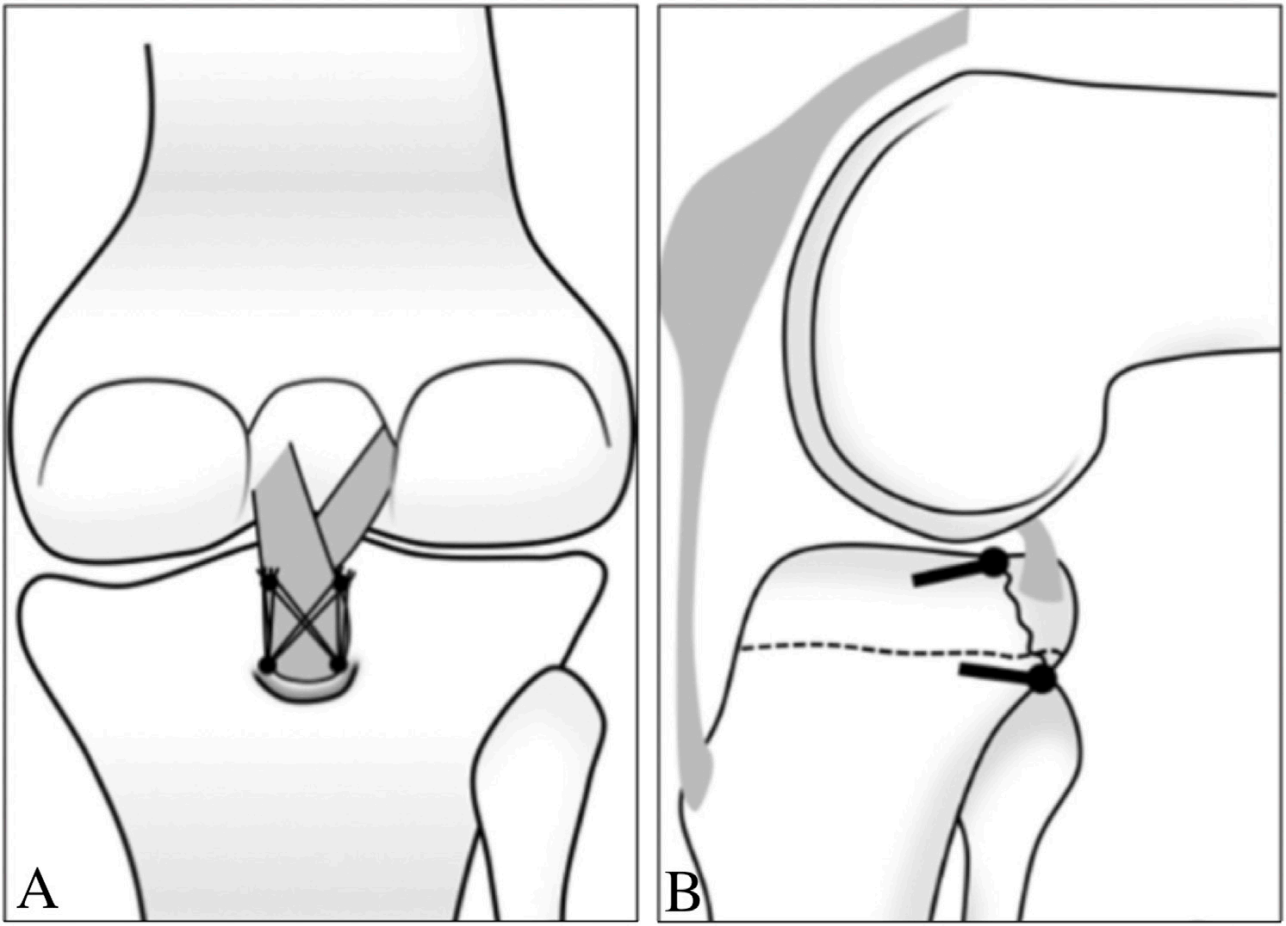
Schematic draw of the suture bridge fixation of PCLAF (Lee et al., 2015) (Copyright 2015; The Korean Orthopaedic Association). **(A)** 2 bio-absorbable anchors were implanted into the proximal-medical side and proximal-lateral side of the fracture bed, and the fragment of PCLAF was fixed by threading the 4 strands of the suture through the bone-tendon junction from anterior to posterior, and the 4 strands (2 strands of each anchor) were retrieved and located by external anchors; **(B)** lateral view, the internal anchors were located proximally, and the external row anchors were located distally of the fracture bed.

Bio-absorbable anchors (4.5 mm Healix BioCryl Rapide Suture Anchors, Depuy Mitek, Johnson & Johnson, Shanghai) was implanted superiorly to the fracture bed. The fragment of PCLAF is fixed using the suture bridge by threading the 4 strands of the suture through the bone-tendon junction from anterior to posterior (Figure 1A). After reducing the fragment by flexion at 90°, the external row anchor (5.5 mm, Healix Advance BioCryl Rapide Knot Free Suture Anchors, Depuy Mitek, Johnson & Johnson, Shanghai) was located at 1.5 cm proximally from the posterior edge of the fracture bed. The 4 strands were retrieved and passed through the external anchor, which was screwed into the bone until enough depth, creating a “suture bridge” fixation (Figure 1B). When the operation was completed, the patient’s limb was placed in a long leg brace with a small pillow under the lower leg to support the lower leg against gravity (Nourbakhsh et al., 2016).

In the control group, the arthroscope was transferred to AM portal, and a Lasso and 2 sutures (no.2 Ethibond suture, Johnson & Johnson, United States) were used to do 2 figure-of-eight sutures through the bone-tendon junction. A 3 cm incision was made on the proximal tibia, which was 2 cm medial to the tibial tuberosity. Transferred the arthroscope to low PM portal, passed a PCL guide through AM portal and space between PCL and medial femur condyle, located it at the medial and lateral side of the fracture bed, and then drilled 2 transtibial tunnels. A beath pin with looped PDS was passed through the tunnels and retrieved with the 2 strands of sutures through the high PM portal, and then the strands were pulled out through the tunnels when reduced the fragment by flexion at 90° (“pulled out” technique). The 4 strands of 2 sutures were tied over a suture disc after maximum pulling and giving anterior drawer at the same time.

### 2.3 Follow-ups

The follow-up was started when the operation was completed. The end was re-operation of the PCL/re-rupture of the PCL/death/missing, whichever occurred first. The minimum follow-up was 1 year (y). Basic clinical parameters included: age, gender, body mass index (BMI), operation time, follow-up time, and complications.

Osseous union was assessed by knee CT scan at 3 months of the follow-up (Lamoria et al., 2020), and knee MRI was performed at 1 year follow-up. All of those data were checked and entered into a database by two researchers, and a double-entry is carried out for quality control.

### 2.4 Clinical assessments

The clinical examinations of PCLAF included the post drawer test (PDT) and gravity test (GT), which were used to assess the anterior-posterior (A-P) joint stability. PDT was classified as: normal (-, posterior shift< 5 mm), doubtable laxity (±, posterior shift between 5 and 10 mm), and laxity (+, posterior shift >10 mm). GT was classified as: normal (-), subsided tibial tuberosity (+). The clinical examinations were performed and recorded before the operation (under anesthesia) and at 1 year follow-up.

A-P laxity (backward shift) was measured by KT-2000 when the knee is flexed at 70°, and it is evaluated by comparing it to the healthy side, and it is classified as (Ranger et al., 2011): normal, grade 1 (difference between 1 and 5 mm), grade 2 (between 5 and 10 mm), and grade 3 (>10 mm). The KT-2000 exam was performed in pre-operation and at 1 year follow-up.

Knee range of motion (ROM) was measured by standardized goniometry technique in pre-operation and at 1 year follow-up. The knee flexion contracture (KFC) and knee flexion limitation (KFL) angles were assessed by passive physical examination of ROM. KFC is defined as the gap value of extension loss compared to the normal side, and KFC ≤5° is normal (Insall et al., 1989; Yi et al., 2020). KFC is classified as: grade 1 (KFC between 5° and 10°), grade 2 (moderate, between 10° and 15°), grade 3 (severe, between 15° and 20), grade 4 (very severe, KFC >20°), according to the Knee Society Score (KSS) system (Insall et al., 1989; Yi et al., 2020). KFL is defined as the gap value of flexion loss compared to the normal side, and KFL is classified as: grade 1 (mild, KFL between 5° and 10°), grade 2 (moderate, between 10° and 15°), grade 3 (severe, between 15° and 20), and grade 4 (very severe, KFL >20°).

### 2.5 Subjective assessments of knee function

To evaluate the functional outcomes of motor function, the Lysholm knee scoring scale (Wang et al., 2016), and International Knee Documentation Committee (IKDC) subjective-form score (Fu and Chan, 2011) were assessed by self-questionnaires at follow-ups. The IKDC score gives equal evaluations of the knee function, while the Lysholm score gives more points to the pain and instability (Lamoria et al., 2020). The full scores of IKDC and Lysholm are 100, and a higher score represents a better functional outcome. The rate of returning to sports was used to evaluate the outcome of physical activity.

### 2.6 Clinical failure

Clinical failure was judge as meeting any of the following results at 1 year follow-up: 1) the re-rupture; 2) overall IKDC score of grade C (60–70 score) or D (less than 60 scores) (Su et al., 2020); 3) PDT (+) or GT (+); 4) A-P laxity of grade 2 or 3 (Fu and Chan, 2011); 5) KFC>5 (grade 1) or KFL>15 (grade 3) (Su et al., 2020). The clinical failure rates were calculated.

### 2.7 Statistical analysis

The continuous data were expressed as mean ± SD, and the inter-group comparisons and intra-group comparisons of the continuous data were processed by the independent samples t-tests and Levene variance homogeneity tests between groups. Count data were expressed as number (n) and rate (/), and inter-group comparisons and intra-group comparisons of the count data were processed by the Chi-square test or Fisher’s exact test. The level of significance was set at 0.05. All of the statistical analyses were performed using SPSS 20.0 (SPSS Inc., 2009; Chicago, IL, United States).

## 3 Results

### 3.1 Basic characteristics

36 patients were enrolled in this study initially, and the sample size of the two groups was set at 1:1. Finally, 31 patients had accomplished the 1 year follow-up, 1 patient of the suture anchor group and 4 patients of the control group were missing, and the total missing rate was 13.9% (5/36). Those missing subjects were excluded from the database, in order to control the bias. Most of the included patients (*n* = 31) were injured by sports or traffic accidents: 13 subjects suffered a sprain of the knee when doing competitive sports; 10 were caused by traffic accident; 2 sprained the knee by themselves when skiing or skating; 4 slipped and sprained the knee by themselves.

The general characteristics including age, gender, BMI, as well as the fracture type, and follow-up time between the suture anchor group (*n* = 17) and control group (*n* = 14) did not have significance (Table 1), while the operative time of the bio-absorbable anchor group was shorter than the control group (Table 1). We did not face any complications such as neurovascular injury, fever, or infection during the perioperative period.

**TABLE 1:** Basic characteristics of PCLAF patients in the bio-absorbable suture anchor and control groups.

Characteristics	Suture anchor (*n* = 17)	Control (*n* = 14)	*p*-value
PCLAF type (partial/complete/comminute)	4/10/3	4/8/2	*χ* ^2^ = 0.133
			*p* = 0.936
Gender (male/female)	11/6	9/5	*χ* ^2^ = 0.001
			*p* = 0.981
Age (year)	24.6 ± 6.3	26.0 ± 6.0	*t* = -0.636
			*p* = 0.530
BMI	22.39 ± 1.19	22.76 ± 1.28	*t* = -0.825
			*p* = 0.416
Operation time (minute)	97.6 ± 13.4	142.6 ± 9.9	*t* = -10.423
			*p* < 0.001**
Follow-up (month)	13.6 ± 2.1	13.4 ± 1.6	*t* = 0.414
			*p* = 0.682

Note: PCLAF (posterior cruciate ligament avulsion fracture); BMI (Body Mass Index); *p* < 0.001**.

### 3.2 Comparisons of the efficacy of the two groups

At 1 year follow-up, the positive rates of PDT and GT, as well as the grade of A-P laxity, KFC, and KFL did not have significance between the bio-absorbable suture anchor group and control group, however, the total failure rate of suture anchor group (1 case: A-P laxity of grade 2) was lower than the control group (3 case: A-P laxity of grade 2–3, 1 case of KFC of grade 1, 1 case of KFL of grade 3) (Table 2).

**TABLE 2:** The efficacy of PCLAF patients in the bio-absorbable suture anchor and control groups at 1 year follow-up.

Parameters		Suture anchor (*n* = 17)	Control (*n* = 14)	*p*-value
PDT	−	15	8	*χ* ^2^ = 4.165
	±	2	5	*p* = 0.125
	+	0	1	
GT	−	17	12	*χ* ^2^ = 2.596
	+	0	2	*p* = 0.107
A-P laxity	Normal	13	7	
	Grade 1	3	4	*χ* ^2^ = 3.206
	Grade 2	1	2	*p* = 0.361
	Grade 3	0	1	
KFC	Normal	17	13	
	Grade 1	0	1	*χ* ^2^ = 1.225
	Grade 2	0	0	*p* = 0.263
	Grade 3	0	0	
	Grade 4	0	0	
KFL	Normal	13	7	
	Grade 1	4	4	*χ* ^2^ = 4.552
	Grade 2	0	2	*p* = 0.208
	Grade 3	0	1	
	Grade 4	0	0	
Total failure rate		1/17	5/14	*χ* ^2^ = 4.377
				*p* = 0.036*

Note: PDT (post drawer test); GT (gravity test); A-P (anterior-posterior), KFC (knee flexion contracture); KFL (knee flexion limitation); *p* < 0.05*.

**FIGURE 2:**
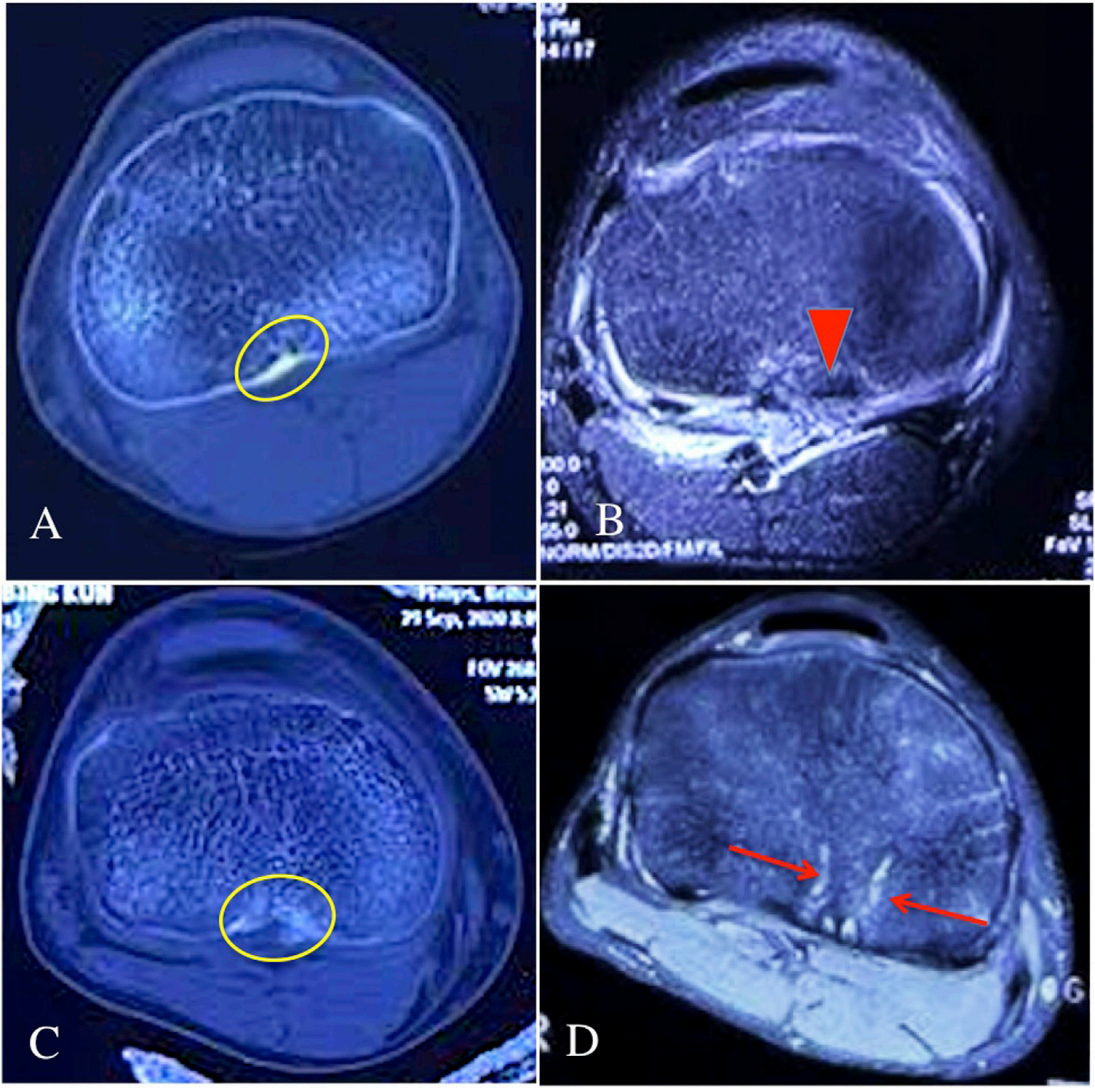
The CT scan and knee MRI of the two groups during the follow-ups. **(A)** knee CT scan of the bio-absorbable group at 3 months follow-up, the yellow circle showed a well bone union of the PCLAF in post-operation; **(B)** knee MRI of the bio-absorbable group at 1 year follow-up, the red triangle showed an intact PCL with the normal signal; **(C)** knee CT scan of the suture pull-out group at 3 months follow-up, the yellow circle showed a well bone union of the PCLAF in post-operation; **(D)** knee MRI of the suture pull-out group at 1 year follow-up, the red arrows showed the trans-tibial tunnels still existed.

CT scan showed that all of the patients in the bio-absorbable group and control group had a well bone union of the PCLAF at 3 months in post-operation (Figures 2A,C). At 1 year follow-up, knee MRI showed that the patients in the bio-absorbable group had an intact PCL with the normal signal (Figure 2B), while the trans-tibial tunnels were still found in the control group (Figure 2D). We did not face any complications such as the bone un-union or re-rupture during the follow-up period.

### 3.3 The functional outcomes of the two groups at 1 year follow-up

No patients of the two groups had an IKDC or Lysholm score lower than 70 scores at 1 year follow-up, and the Lysholm score did not have significance between the bio-absorbable suture anchor group and control group, but the IKDC of bio-absorbable suture anchor group was higher than that of the control group (Table 3). The return to sports rate in the bio-absorbable anchor group was also higher than that in the control group (Table 3).

**TABLE 3:** The functional outcomes of the bio-absorbable suture anchor and control groups at 1 year follow-up.

Parameters		Suture anchor (*n* = 17)	Control (*n* = 14)	*p*-value
Lysholm		86.88 ± 5.24	86.21 ± 3.26	*t* = 0.415
				*p* = 0.681
IKDC		86.24 ± 4.35	81.43 ± 5.06	*t* = 2.843
				*p* = 0.008**
Return to sports	Yes	11	4	*χ* ^2^ = 4.014
	No	6	10	*p* = 0.045*

Note: *p* < 0.01**; *p* < 0.05*.

## 4 Discussion

### 4.1 PCLAF and sports-injury in the young population

PCLAF is a special type of PCL rupture, and it can occur in the setting of high-energy trauma such as motorcycle accidents, as well as in lower-energy sports-related injuries in young and active individuals (Schulz et al., 2003). However, the present study found that most of the included patients were injured by sports (13 cases of competitive sports, 2 cases of skiing or skating), high-energy trauma was ranked second (10 cases of traffic accidents), followed by lower-energy trauma (4 cases of slip and self-sprain). In our study, the included patients were young population with the age ranging from 18 to 38, who were physically active and prone to get injured during sports. The relatively young population may explain the reason why the lower-energy sports-related injuries ranked first in the PCLAF patients.

### 4.2 Arthroscopic fixations for PCLAF

PCLAF always needs surgical fixation in order to achieve sufficient knee stability and adequate bone healing (Willinger et al., 2019). Although it has been considered that a fragment with a displacement of more than 5 mm is an operative indication (Zhao et al., 2006), the failure rate of conservative treatment for PCLAF is relatively high as a matter of fact. Because the tibial insertion of PCL is located outside the capsule, the surrounding soft tissue of PCLAF is easy to be embedded into the fracture, causing non-union. Therefore, in the present study, we performed the arthroscopic fixation for all the patients, including PCLAF with the partial, complete, or comminuted fragment.

A whole arthroscopic fixation of PCLAF has been firstly presented by Dr. Dinshaw Pardiwala in 2009, until now, several arthroscopic techniques have been described for fixation of PCLAF, and which have been classified into the screw-based, anchor-based, and pull-out techniques (Lamoria et al., 2020). Correspondingly, there is a wide variety of biomaterials available for arthroscopic fixation of PCLAF, for example, the absorbable interference screw (Li et al., 2016), polyether-ether-ketone (PEEK)-based suture anchor, and absorbable polymer-based suture anchor. The screw-based PCLAF fixation has a series of disadvantages, which includes a risk of epiphyseal damage and destruction of the avulsed fragment, hence, it is not suitable for the PCLAF with a small fragment (Willinger et al., 2019). Therefore, the suture-bridge fixation based on suture anchors was developed to fixate the small and multi-fragment fractures, without the need for fragment drilling (Willinger et al., 2019), and it is possible to reduce the risk of bone fragment destruction (Kanayama et al., 2022). The first article about the arthroscopic suture bridge technique for PCLAF fixation has been reported in 2016 (Nourbakhsh et al., 2016), although it has been commonly used in the field of shoulder arthroscopy for many years. The pull-out technique is a traditional arthroscopic fixation method of PCLAF, similar to the suture bridge, it is not limited to the conditions of fragments either (Willinger et al., 2019) (Nakagawa et al., 2017). Hence, we compared the efficacy and outcomes of the bio-absorbable suture anchor with the pull-out technique on treatment of PCLAF, as both the two arthroscopic techniques have a general clinical application (Willinger et al., 2019) (Nakagawa et al., 2017).

### 4.3 Safety, complication, and bone union

The present compared the safety, complications, and bone union of the two arthroscopic fixation methods for PCLAF with or without the bio-absorbable fixation materials. Our results showed that no complication of neurovascular injury, fever, and infection was found during the perioperative period. It suggests that both of the arthroscopic fixation methods had superior safety. It has been generally recognized that these arthroscopic methods are safer and less invasive than the posterior open approach for PCLAF fixation (Nakagawa et al., 2017), which requires a large skin incision to avoid damage to the popliteal neurovascular structures located immediately behind the site (Nakagawa et al., 2017), and has a risk of neurovascular injury (Lopez-Vidriero et al., 2010). However, the operation time of the suture bridge was much shorter than the traditional pull-out technique (97.6 ± 13.4 vs. 142.6 ± 9.9 min), hence, the suture bridge fixation for PCLAF may have more benefits in decreasing the peri-operation complication than the traditional technique.

Our results showed that both of the arthroscopic fixation groups did not have complications of bone un-union or re-rupture during the follow-up. It indicates that the arthroscopic fixation techniques can provide enough strength for the fixation and bone union of the avulsion fragment. We performed a double-row suture bridge in the bio-absorbable anchor group, similar to our results, Kanayama et al. found that PCLAF fixation by suture bridge can firmly fix the bone fragments, and it is supposed to reduce the risk of fragment destruction (Kanayama et al., 2022), such as non-union and re-rupture. We performed a pull-out fixation with Ethibond suture in the control group, and a biomechanical study has proved that the mechanical properties of the Ethibond suture were comparable with the screw fixation on tibial eminence fractures, in terms of cyclic load, stiffness, and maximum load (Eggers et al., 2007). The present study suggests that both the bio-absorbable suture anchor and pull-out technique on fixation of PCLAF can provide a rigid fixation and sufficient strength for the reduction and bone union.

### 4.4 Efficacy and knee functional outcomes

The present compared the efficacy, clinical failure rate, and complications, and the functional outcomes of the bio-absorbable anchor and pull-out technique for PCLAF fixation. Our results found that although the positive rates of PDT and GT, as well as the grade of A-P laxity, KFC, and KFL, did not have a significant difference between the two groups, the total failure rate of the suture anchor group (1/17) was significantly lower than the control group (5/14) at 1 year follow-up. In our study, the patient with PDT (+)/GT (+), A-P laxity≥2 (Su et al., 2020), KFC>5 (grade 1) (Su et al., 2020), and KFL>15 (grade 3) (Su et al., 2020) were considered as the clinical failure case, as those were important indicators for assessing knee stability and ROM, and which were closely associated with the patient’s functional outcomes. What’s more, our study also found that the IKDC and return to sports rate in the bio-absorbable anchor group were higher than the control group. IKDC is more sensitive compared to Lysholm score while evaluating knee ligament injuries (Farshad et al., 2011), and return to sports rate is the ultimate indication for evaluating the functional outcomes. Our results indicate that arthroscopic fixation of PCLAF with the bio-absorbable suture anchor has better efficacy and functional outcomes than the pull-out suture fixation.

The bio-absorbable suture anchor has less impact on the tibial cancellous bone and blood supply, which can explain the reason why it can acquire better efficacy and functional outcomes than the pull-out suture fixation. The local blood supply may be more important in patients with PCLAF. Patients with PCLAF may have a relatively weaker bone structure in comparison with the PCL, which makes they are prone to having a PCLAF rather than a PCL rupture (Kanayama et al., 2022). The pull-out suture fixation was based on a trans-tibial tunnel, which results in a bony defect and destruction of the local blood supply for a long period post-operatively. The local blood supply is extremely important to ligament recovery, for example, the stumps are always preserved as much as possible in ACL and PCL reconstruction, in order to preserve the blood supply (Yuanliang et al., 2020). Our results have shown that the trans-tibial tunnel and bony defect can still be found on 1 year post-operative MRI in the pull-out group, and it can be adverse to the local blood supply, as well as the recovery process of PCL. Similar to our results, a follow-up study reported the efficacy of the trans-tibial pull-out fixation for PCLAF (same with ours, trans-tibial cortical suspension with suture and suture disc), they found that 2 patients (2/22) had a grade 2 laxity (5–10 mm) at 1 year post-operatively, and 3 patients had a complication of KFL who eventually ended up with 110–120 of flexion (Lamoria et al., 2020). As the suture bridge fixation technique did not need the tunnel (Willinger et al., 2019), it is supposed to acquire a better efficacy and outcomes than the traditional pull-out technique. Kanayama et al. used the suture bridge technique for PCLAF open fixation, finally, no perioperative complication was found, and all patients returned to full sporting activity (Kanayama et al., 2022). On the other hand, the bio-absorbable suture anchor consists of 70% of polylactic acid-glycolic acid copolymer (PLGA, 85% L-lactic acid, 15% glycolic acid), and 30% of β-tricalcium phosphate, which have superior *in-vivo* safety, without impact on local cancellous bone and blood supply (Koch et al., 2021). Hence, the bio-absorbable suture anchor for arthroscopic fixation of PCLAF can result in a well-recovered PCL with the least impact on the blood supply.

The biomechanical mechanism may be another potential mechanism that explains the reason why the bio-absorbable suture anchor can acquire better efficacy and outcomes. Many studies have reported that fixation strength produced by suture-bridge is greater than pull-out suture fixation. A biomechanical comparison study by Willinger et al. found that the suture-bridge technique for PCLAF fixation resulted in a significant lower elongation (4.5 ± 2.9 mm) than the transtibial pull-out technique (11.9 ± 3.1 mm) during cyclic loading, suggesting that PCLAF with suture bridge can fix the bone fragments more firmly than the traditional pull-out technique (Willinger et al., 2019). The PCL post-operative elongation is associated with poor knee stability and physical function, which may contribute to the low IKDC and return to sports rate in the suture pull-out group.

Our study had several limitations. First, it was not an RCT study, which cannot avoid the selected bias. Second, our sample size was relatively small, and the follow-up time did not reach the long-term. As the present study was a pilot study, further RCTs and longitudinal studies with more samples and longer follow-up are needed as well as to explore and determine the long-term outcome of using bio-absorbable anchors for PCLAF fixation.

## 5 Conclusion

Both the bio-absorbable anchor and suture pull-out technique for arthroscopic fixation of PCLAF had superior perioperative safety and a well bone union at 3 months in post-operation. However, 1 year follow-up showed that the bio-absorbable anchor group had better efficacy and functional outcomes than the traditional suture pull-out technique, suggesting the novel bio-absorbable anchor has a wide clinical application prospect on treatment of PCLAF.

## Data Availability

The original contributions presented in the study are included in the article/Supplementary Material, further inquiries can be directed to the corresponding author.
